# Steady-State Thermal Analysis of Functionally Graded Rotating Disks Using Finite Element and Analytical Methods

**DOI:** 10.3390/ma15165548

**Published:** 2022-08-12

**Authors:** M. M. Shahzamanian, A. Shahrjerdi, B. B. Sahari, P. D. Wu

**Affiliations:** 1 Department of Mechanical Engineering, McMaster University, Hamilton, ON L8S 4L7, Canada; 2 Department of Mechanical Engineering, Texas A&M University, College Station, TX 77843-3123, USA; 3 Mechanical Engineering Department, Malayer University, Malayer, Iran; 4 Faculty of Engineering and Built Environment, UCSI University, Kuala Lumpur 56000, Malaysia

**Keywords:** finite element method (FEM), functionally graded materials (FGMs), steady-state thermal analysis

## Abstract

A steady-state thermal analysis for a hollow and axisymmetric functionally graded (FG) rotating disk with a uniform thickness was performed in this study. In the studied FG disk, metal and ceramic materials were considered for the inner and outer surfaces, respectively, when the material properties varied along the radial direction but not through material thickness variations. A power law distribution was employed to represent the material properties. Three different methods were used to present the temperature distribution along the radial direction of the FG disk, namely (1) an in-house finite element (FE) program, (2) the ANSYS parametric design language (APDL), and (3) an analytical solution. Furthermore, the in-house FE program presented the thermal stress and thermal strain of the FG disk. The weighted residual method in the FEM was used to present the temperature distribution when the material properties along an element are varying in contrast with using a commercial finite element software when the material properties are constant within an element to simulate FGMs. The accuracy of the in-house FE program was tested, and it was shown that the temperature distributions obtained by using the abovementioned methods were exactly the same. A parametric material gradation study was performed to understand the effects on the temperature, thermal strain, and stress. The material gradation was found to have a significant effect in this regard. The in-house finite element program enables one to perform a post-processing analysis in a more efficient and convenient manner than that through simulations in a finite element software program such as ANSYS. Lastly, this in-house code can be used to perform an optimization analysis to minimize the thermal strain and stress while the stiffness of the plate is maintained when the material properties within an element vary.

## 1. Introduction

**Figure 1:**
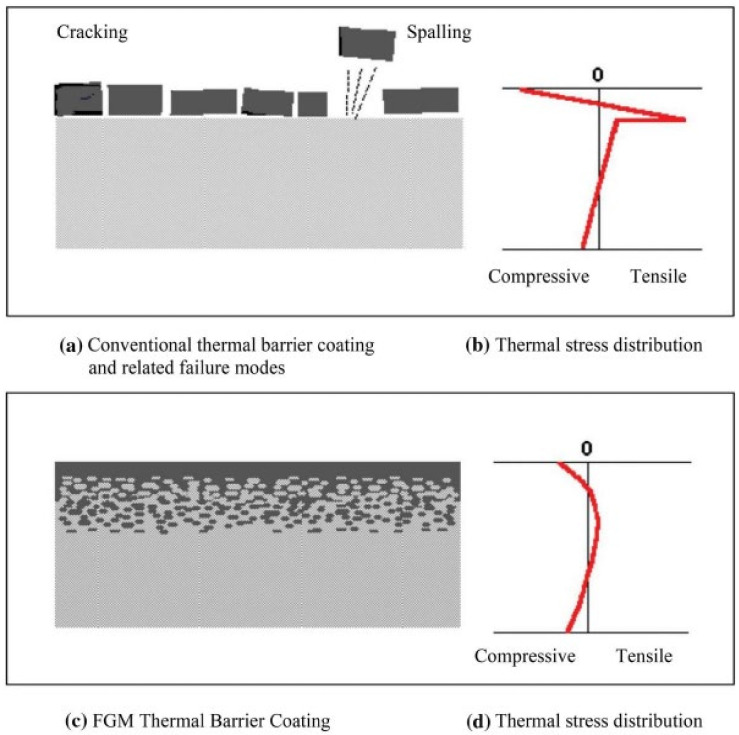
Comparison between a coated material and FGM used to resist thermal loads [5].

The material properties vary smoothly and continuously based on a specified function along direction(s) in functionally graded materials (FGMs) [1,2,3]. Thus, the thermal and mechanical properties can vary from metals to ceramics in FG rotating disks. FGMs are mainly fabricated to operate in high-temperature environments such as in ultra-light, temperature-resistant materials used for space vehicles [4]. FGMs offer an advantage because the thermal stresses are smooth compared with when the disk is coated [5] (Figure 1a,b for a coated disk and Figure 1c,d for an FGM). As such, the steady-state temperature distributions in FG rotating disks are analyzed in this study. The results presented here were obtained using the finite element method (FEM), the commercial software ANSYS, the in-house written code in MATLAB, and an analytical method. The material properties along an element vary using the in-house FE code in contrast with using the commercial FE software when the material properties are constant within an element when simulating FGMs.

Many researchers have performed thermoelastic analyses on FG rotating disks using analytical methods [6,7,8,9,10,11,12,13,14,15]. Bayat et al. [6,7] performed a thermoelastic analysis of FG rotating disks considering the small and large deflections under constant and variable thicknesses. In another study by Jabbari et al. [8,9], a thermoelastic analysis of short and long thick hollow FG cylinders was performed. Zenkour [10] performed a numerical analysis of the stress distribution of the rotating composite structures of FG disks. Eldeeb et al. [11] performed a thermoelastic analysis for a rotating sandwich disk made of temperature dependent FGMs. Erdogan and Wu [12] analyzed the crack problems for FGMs under thermal stresses. Duc et al. [13] performed an analysis to investigate the non-linear dynamic response of shear deformable FGM plates. In all mentioned works, the analytical methods were found useful in analyzing the responses of FGMs and in investigating the effect of material gradation under various conditions.

The FEM is an applicable tool for simulating FGMs and analyzing their responses under various conditions [16,17,18,19,20,21,22,23,24,25,26]. FG rotating disks can be simulated using the FEM to analyze the thermomechanical results [18,19,20,21]. FGMs have been simulated using the FEM by Durodola and Attia [22], and the deformation and stresses have been analyzed. Shahzamanian et al. [23,24,25] performed a simulation for FG brake disks using the APDL. The material properties of FG brake disks vary in radial [23] and thickness [25] directions. The thermomechanical results of FG brake disks under pressure and heat sources due to friction between the pads and disks have been analyzed. The effects of various contact parameters in FG brake disks were studied in [24]. Genao et al. [26] used a non-linear FEM to perform a thermoelastic analysis of temperature dependent FGMs. Overall, the material gradation has been found to have a significant effect on the thermomechanical responses of FGMs [27,28,29,30,31].

Steady-state thermal analyses of FGMs have been performed by many researchers. Functionally graded materials (FGMs) can resist high temperatures and can reduce the thermal stresses [32]. Jabbari et al. [33,34] presented the steady-state thermal and mechanical stresses for functionally graded piezo-electric porous material (FGPPM) hollow spheres. Bayat et al. [35] performed an analytical and numerical analysis to present the thermal and mechanical response of an FGM under combined pressure and temperature loading. Delouei et al. [36] presented the steady-state two-dimensional temperature distribution for an FGM. In their analysis, a Fourier transform was used.

In the present study, an FG hollow rotating disk was considered, and the material properties of the FG rotating disk were assumed via the power law distribution with respect to the radial direction. The inner and outer surfaces were completely metal and ceramic, respectively. The temperature distribution of the FG disks with various material gradations was calculated and presented using three methods: a simulation in ANSYS software, an analytical solution, and an in-house FE program. The thermal stress and thermal strain of the FG rotating disk were determined using the in-house FEM. The results using the ANSYS software were verified, and a parametric study was performed to understand the effects of each material gradation on the temperature distribution, thermal strain, and stress distributions. The in-house finite element code makes the post-processing analysis more convenient and efficient than that performed using ANSYS software.

## 2. Material Gradation Relation

In this study, as mentioned previously, the material properties of the FG disk vary along the radial direction using the power law distribution [8]:

(1) $$P\left(r\right)\;=\;P_{•}r^{m}$$

where P is the material property at radius r and $P_{•}$ and m are material constants. $E_{•}$, $\alpha_{•}$, and $K_{•}$ are constant parameters for the Young’s modulus, thermal expansion coefficient, and thermal conductivity, respectively to create the material variation in FG disks. Moreover, $m_{\mathrm{YM}}$, $m_{\mathrm{TE}}$, and $m_{\mathrm{TC}}$ are the power parameters for the Young’s modulus, thermal expansion, and thermal conductivity, respectively.

## 3. Methodology and Problem Formulation

As mentioned previously, first the temperature distribution of the FG rotating disk was presented for validation and accuracy testing in to present the thermoelastic results for the three different methods. The three different methods are as follows [37]:

1. In-house finite element program;
2. APDL;
3. Analytical solution.

In this section, each method will be described in detail and in a stepwise manner. We describe how these methods calculate the temperature distribution of an FG disk when all material properties, such as the Young’s modulus, thermal conductivity, and thermal expansion coefficient, vary along the radius. The thermal conductivity varies along an element using the in-house FE code in contrast with using the FE commercial software when the thermal conductivity is constant within an element to simulate FGMs.

### 3.1. Method #1: In-House FE Program

In an FG disk, the temperature distribution can be stated as [38,39]:

(2) $$\frac{1}{r}\left(\frac{\partial }{\partial r}\right)\left(\mathrm{rk}\left(r\right)\frac{\partial T}{\partial r}\right)\;+\;\frac{\partial }{\partial z}\left(k\left(r\right)\frac{\partial T}{\partial z}\right)\;=\;0$$

The following expressions are given by expanding Equation (2):

(3) $$\left(\left[\frac{k\left(r\right)}{r}\;+\;k\prime \left(r\right)\right]\frac{\partial T}{\partial r}\;+\;k\left(r\right)\frac{\partial^{2}T}{\partial r^{2}}\right)\;+\;k\left(r\right)\frac{\partial^{2}T}{\partial z^{2}}\;=\;0$$

(4) $$\left[\frac{1}{r}\;+\;\frac{k\prime \left(r\right)}{k\left(r\right)}\right]\frac{\partial T}{\partial r}\;+\;\frac{\partial^{2}T}{\partial r^{2}}\;+\;\frac{\partial^{2}T}{\partial z^{2}}\;=\;0$$

The following equation is obtained using Equation (1):

(5) $$\frac{k\prime \left(r\right)}{k\left(r\right)}\;=\;\frac{m_{\mathrm{TC}}}{r}$$

The following equations are obtained by substituting Equation (5) into Equation (4):

(6) $$\left[\frac{1}{r}\;+\;\frac{m_{\mathrm{TC}}}{r}\right]\frac{\partial T}{\partial r}\;+\;\frac{\partial^{2}T}{\partial r^{2}}\;+\;\frac{\partial^{2}T}{\partial z^{2}}\;=\;0$$

(7) $$\frac{\partial^{2}T}{\partial r^{2}}\;+\;\frac{1}{r}\frac{\partial T}{\partial r}\;+\;\frac{\partial^{2}T}{\partial z^{2}}\;+\;\left(\frac{m_{\mathrm{TC}}}{r}\right)\frac{\partial T}{\partial r}\;=\;0$$

The element matrix integral is calculated by applying the weighted residual method [40]:

(8) $$\int_{\Omega }\omega \left(\frac{\partial^{2}T}{\partial r^{2}}\;+\;\frac{1}{r}\frac{\partial T}{\partial r}\;+\;\frac{\partial^{2}T}{\partial z^{2}}\;+\;\left(\frac{m_{\mathrm{TC}}}{r}\right)\frac{\partial T}{\partial r}\right)d\Omega$$

where “ω” is the weighted residual. Equation (8) can be rewritten as follows:

(9) $$\int_{\Omega }\omega \left(\frac{1}{r}\frac{\partial }{\partial r}\left(\frac{\partial T}{\partial r}\right)\;+\;\frac{\partial^{2}T}{\partial z^{2}}\;+\;\left(\frac{m_{\mathrm{TC}}}{r}\right)\frac{\partial T}{\partial r}\right)d\Omega$$

Thereafter, the domain integral can be expressed as an axisymmetric cylindrical, as shown in Equation (10):

(10) $$\begin{array}{l} \int_{\Omega }\omega \left(\frac{1}{r}\frac{\partial }{\partial r}\left(\frac{\partial T}{\partial r}\right)\;+\;\frac{\partial^{2}T}{\partial z^{2}}\;+\;\left(\frac{m_{\mathrm{TC}}}{r}\right)\frac{\partial T}{\partial r}\right)d\Omega \\ \;=\;2\pi \int_{r}\int_{z}r\left(\omega \left(\frac{1}{r}\frac{\partial }{\partial r}\left(\frac{\partial T}{\partial r}\right)\;+\;\frac{\partial^{2}T}{\partial z^{2}}\;+\;\left(\frac{m_{\mathrm{TC}}}{r}\right)\frac{\partial T}{\partial r}\right)\right)\mathrm{dr}\;\mathrm{dz} \end{array}$$

Equation (10) is rewritten based on Equation (11):

(11) $$2\pi \int_{r}\int_{z}\omega \left(\frac{\partial }{\partial r}\left(\frac{\partial T}{\partial r}\right)\;+\;r\frac{\partial^{2}T}{\partial z^{2}}\;+\;m_{\mathrm{TC}}\frac{\partial T}{\partial r}\right)\mathrm{dr}\;\mathrm{dz}$$

The weak formulation of the first two terms in Equation (11) using the integration parts is replaced in Equation (12) [40]:

(12) $$2\pi \int_{r}\int_{z}r\left(\;-\;\frac{\partial \omega }{\partial r}\frac{\partial T}{\partial r}\;+\;\frac{\partial \omega }{\partial z}\frac{\partial T}{\partial z}\;+\;\frac{m_{\mathrm{TC}}}{r}\frac{\partial T}{\partial r}\right)\mathrm{dr}\;\mathrm{dz}$$

**Figure 2:**
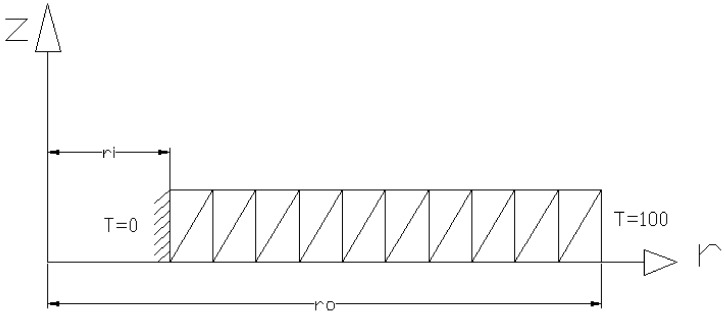
Element distribution of the FG disk using an axisymmetric triangular element.

In the in-house FE method, 80 axisymmetric triangular elements were used. A mesh study was performed and the number of elements was selected in order to not have a significant effect on the results, and in the meantime to minimize the computational effort. Figure 2 shows the mesh division by the triangular elements.

For the axisymmetric triangular element, the element matrix is stated as presented in Equation (13).

(13) $$\left[k\right]\;=\;2\pi \int_{z}\int_{r}\left\{r\left(-\left\{\begin{array}{c} \frac{\partial H_{1}}{\partial r}\\ \frac{\partial H_{2}}{\partial r}\\ \frac{\partial H_{3}}{\partial r} \end{array}\right\}\left\{\begin{array}{ccc} \frac{\partial H_{1}}{\partial r} & \frac{\partial H_{2}}{\partial r} & \frac{\partial H_{3}}{\partial r} \end{array}\right\}\;-\;\left\{\begin{array}{c} \frac{\partial H_{1}}{\partial z}\\ \frac{\partial H_{2}}{\partial z}\\ \frac{\partial H_{3}}{\partial z} \end{array}\right\}\left\{\begin{array}{ccc} \frac{\partial H_{1}}{\partial z} & \frac{\partial H_{2}}{\partial z} & \frac{\partial H_{3}}{\partial z} \end{array}\right\}\;+\;\frac{m_{\mathrm{TC}}}{r}\left\{\begin{array}{c} H_{1}\\ H_{2}\\ H_{3} \end{array}\right\}\left\{\begin{array}{ccc} \frac{\partial H_{1}}{\partial r} & \frac{\partial H_{2}}{\partial r} & \frac{\partial H_{3}}{\partial r} \end{array}\right\}\right)\right\}\mathrm{dzdr}$$

Shape functions for an axisymmetric triangular element are denoted by $H_{1},H_{2},\;\mathrm{and}\;H_{3}$.

Lastly, Equation (14) is solved to determine the vector of the temperature (T) as follows:

(14) $$\left[K\right]\left\{T\right\}\;=\;\left\{F\right\}$$

In Equation (14), F is a matrix representing the force vector.

A MATLAB code was implemented, and the nodes and elements were created in an algorithm. Then, the element matrix of every element ($\left[k\right]:\mathrm{small}\;“k”$) was calculated, and they were considered in $\left[K\right]$ (Capital ‘‘K’’) to solve Equation (14). The temperature boundary conditions were applied, and finally the temperature distribution was calculated. The implemented in-house code has been shared for public use and can be downloaded from [41].

### 3.2. Method #2: APDL

**Figure 3:**
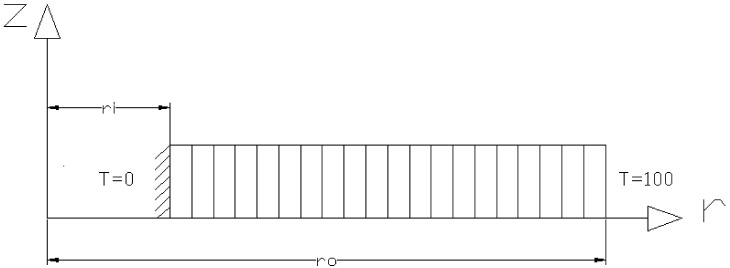
Element distribution of an FG disk using ANSYS software.

For the validation and accuracy testing of method #1, the FG rotating disk was divided into 40 elements along the radius, as shown in Figure 3, using ANSYS. The APDL was used to simulate the FG disk. Again, a mesh study was performed and the number of elements was selected in a manner so as to not have a significant effect on the results, and in the meantime to minimize the computational effort. The Plane42 element in ANSYS was used to obtain the temperature distribution. Shahzamanian et al. [23,24,25] have explained the simulation of FGMs in ANSYS. However, the material properties of the FG rotating disk were calculated at the mean radius of each element. By applying the temperature at the inner and outer surfaces of disks, the temperature distribution will be given by ANSYS.

### 3.3. Method #3: Analytical Solution

An analytical solution also was used to determine the temperature distribution and the obtained results were compared with those presented by the previous methods. In this study, the variation in temperature distribution was only along the radius. Therefore, neglecting the terms in the thickness (z) direction in Equation (2) leads to obtaining Equations (15) and (16).

(15) $$\frac{\partial^{2}T}{\partial r^{2}}\;+\;\left(\frac{m_{\mathrm{TC}}\;+\;1}{r}\right)\frac{\partial T}{\partial r}\;=\;0$$

(16) $$r^{2}\frac{\partial^{2}T}{\partial r^{2}}\;+\;\mathrm{Ar}\frac{\partial T}{\partial r}\;=\;0$$

where, $A\;=\;m_{\mathrm{TC}}\;+\;1$. By considering, $r\;=\;e^{t}\;\mathrm{or}\;t\;=\;\ln\left(r\right)$, the following expressions are presented sequentially:

(17) $$\frac{\mathrm{dT}}{\mathrm{dr}}\;=\;\frac{\mathrm{dT}}{\mathrm{dt}}\frac{\mathrm{dt}}{\mathrm{dr}}\;=\;\frac{\mathrm{dT}}{\mathrm{rdt}}$$

(18) $$\begin{array}{c} \frac{d^{2}T}{{\mathrm{dr}}^{2}}\;=\;\frac{d}{\mathrm{dr}}\left(\frac{\mathrm{dT}}{\mathrm{dr}}\right)\;=\;\frac{d}{\mathrm{dr}}\left[\frac{\mathrm{dT}}{\mathrm{dt}}\frac{1}{r}\right]\;=\;\frac{d}{\mathrm{dr}}\left[\frac{\mathrm{dT}}{\mathrm{dr}}e^{-t}\right]\;=\;\frac{d\left[\frac{\mathrm{dT}}{\mathrm{dr}}e^{-t}\right]}{\mathrm{dt}}\frac{\mathrm{dt}}{\mathrm{dr}}\\ =\;\frac{d\left[\frac{\mathrm{dT}}{\mathrm{dr}}e^{-t}\right]}{\mathrm{dt}}\frac{1}{r}\;=\;\left[\frac{d^{2}T}{{\mathrm{dt}}^{2}}e^{-t}\;-\;\frac{\mathrm{dT}}{\mathrm{dt}}e^{-t}\right]e^{-t} \end{array}$$

By substituting Equations (17) and (18) into Equation (16), one obtains:

(19) $$\begin{array}{c} \frac{d^{2}T}{{\mathrm{dt}}^{2}}e^{-2t}\;-\;\frac{\mathrm{dT}}{\mathrm{dt}}e^{-2t}\;+\;{\mathrm{Ae}}^{-t}\frac{\mathrm{dT}}{\mathrm{dr}}e^{-t}\;=\;\frac{d^{2}T}{{\mathrm{dt}}^{2}}e^{-2t}\;-\;\frac{\mathrm{dT}}{\mathrm{dt}}e^{-2t}\;+\;A\frac{\mathrm{dT}}{\mathrm{dr}}e^{-2t}\\ =\;\frac{d^{2}T}{{\mathrm{dt}}^{2}}e^{-2t}\;+\;\left(A-1\right)\frac{\mathrm{dT}}{\mathrm{dt}}e^{-2t}\;=\;0 \end{array}$$

or Equation (19) can be rewritten as follows:

(20) $$T\prime \prime \;+\;\left(A\;-\;1\right)T\prime \;=\;0$$

(21) $$\frac{d\left(\frac{dT}{dt}\right)}{dt}\;=\;\left(1\;-\;A\right)\frac{dT}{dt}$$

By considering $\frac{\mathrm{dT}}{\mathrm{dt}}\;=\;X$, the following equations are expressed sequentially.

(22) $$X\;=\;e^{\left(1-A\right)t\;+\;c_{1}}$$

(23) $$T\prime \left(t\right)\;=\;e^{\left(1-A\right)t+c_{1}}$$

(24) $$T\left(t\right)\;=\;\frac{e^{\left(1-A\right)t+c_{1}}}{\left(1\;-\;A\right)}\;+\;c_{2}$$

(25) $$T\left(r\right)\;=\;\frac{e^{\left(1-A\right)\ln r+c_{1}}}{\left(1\;-\;A\right)}\;+\;c_{2}$$

Finally, the temperature distribution is:

(26) $$T\left(r\right)\;=\;\frac{e^{-m_{\mathrm{TC}}\ln r\;+\;c_{1}}}{\;-\;m_{\mathrm{TC}}}\;+\;c_{2}$$

where $c_{1}\;\mathrm{and}\;c_{2}$ are constants, which are found by applying the boundary condition.

## 4. Thermal Stress and Strain Relations

The thermal stress and strain were determined using the in-house FE code, and the effect of the material gradation was studied as well. Equations (27) and (28) present the thermal strain and stress in an FG disk, respectively [42].

(27) $$\varepsilon^{T}\;=\;\alpha \left(r\right)T\left(r\right)$$

(28) $$\sigma^{T}\;=\;\frac{E\left(r\right)}{\left(1\;-\;\upsilon^{2}\right)}\left[\left(1\;+\;\upsilon \right)\alpha \left(r\right)T\left(r\right)\right]$$

where $E\left(r\right)\;=\;E_{•}r^{m_{\mathrm{YM}}}$ and $\alpha \left(r\right)\;=\;\alpha_{•}r^{m_{\mathrm{TE}}}$. $\alpha_{•},\;m_{\mathrm{TE}},\;E_{•}\;\mathrm{and}\;m_{\mathrm{YM}}$ are material parameters, and υ is Poisson’s ratio.

## 5. Numerical Results and Discussion

### 5.1. Material Property Variations in an FG Disk

In this case, the values of $\frac{r_{i}}{r_{o}}\;=\;0.2$ and $\frac{r_{i}}{h}\;=\;0.2$ were used, where $r_{i}$ and $r_{o}$ are the inner and outer radii, respectively, and h is the thickness of a hollow FG brake disk. The material properties of the inner and outer surfaces as metals and ceramics are shown in Table 1 [8], and the FG gradation material properties were calculated and are shown in Table 2. Poisson’s ratio was constant in the rotating disk.

**Figure 4:**
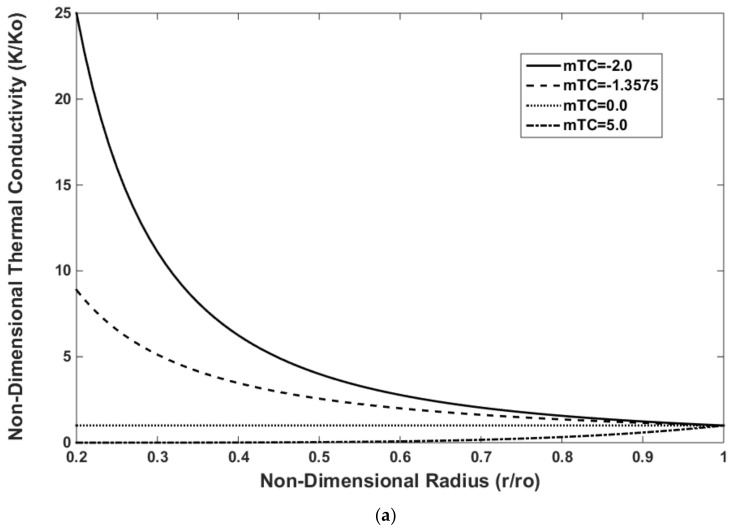

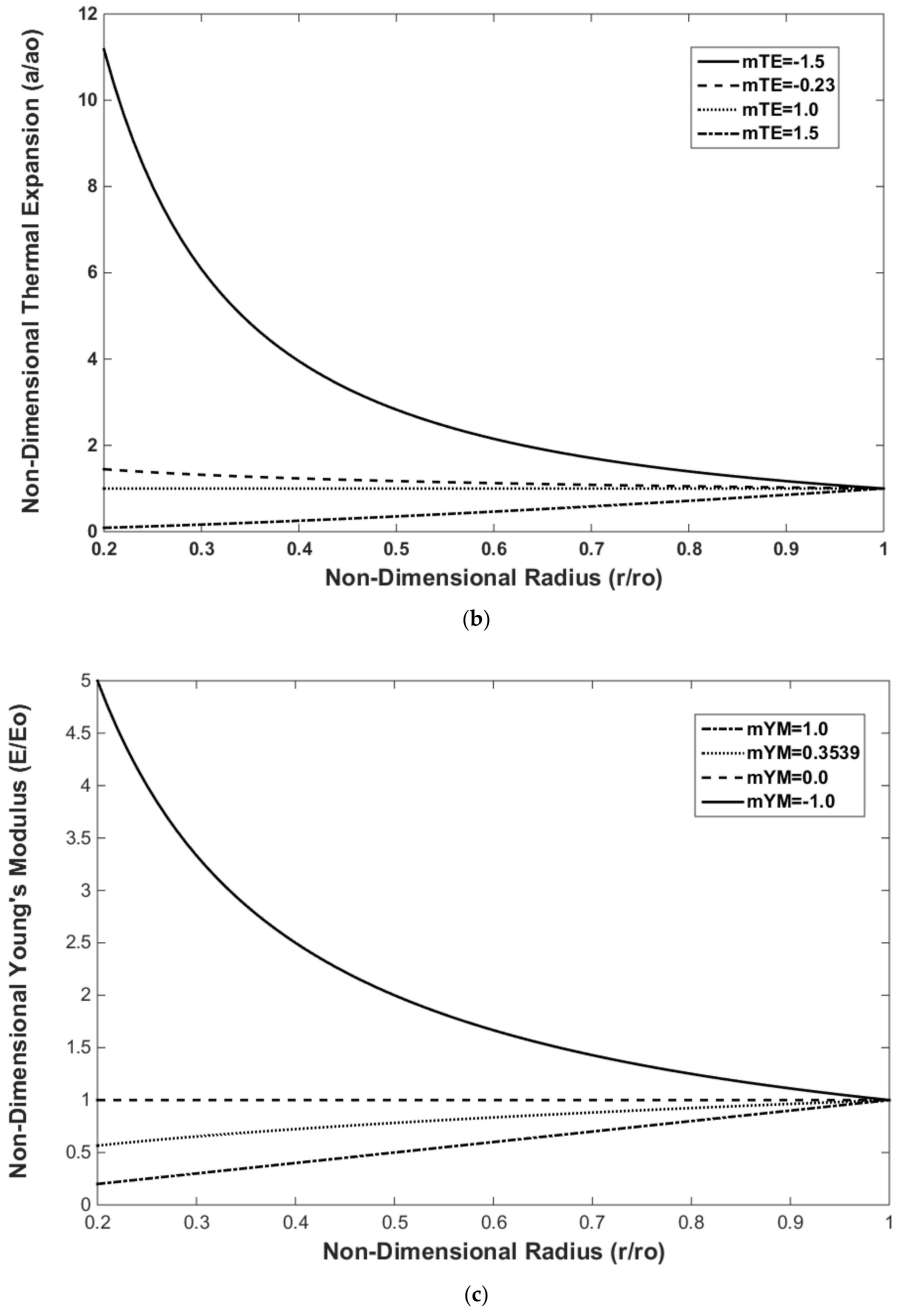
Variations in material properties in FG rotating disks along the radius: (**a**) thermal conductivity; (**b**) thermal expansion; (**c**) Young’s modulus.

Figure 4 shows the variations in non-dimensional material properties along the non-dimensional radius ($r/r_{o}$). The material properties were non-dimensionalized by dividing the material properties at the outer surface of the FG rotating disk. Young’s modulus increases, and the thermal properties decrease along the radius of the disk when $m_{\mathrm{YM}}\;=\;0.3539$, $m_{\mathrm{TE}}\;=\;-0.23$, and $m_{\mathrm{TC}}\;=\;-1.3575$. These values correspond to the FGM used in this study. However, a material gradation study was performed, and the effects of each parameter on the material properties are shown in Figure 4. The effects of $m_{\mathrm{TC}}$, $m_{\mathrm{TE}}$, and $m_{\mathrm{YM}}$ on the temperature distribution, thermal strain, and thermal stress will be discussed later in this section.

### 5.2. Numerical Results of the Three Methods

**Figure 5:**
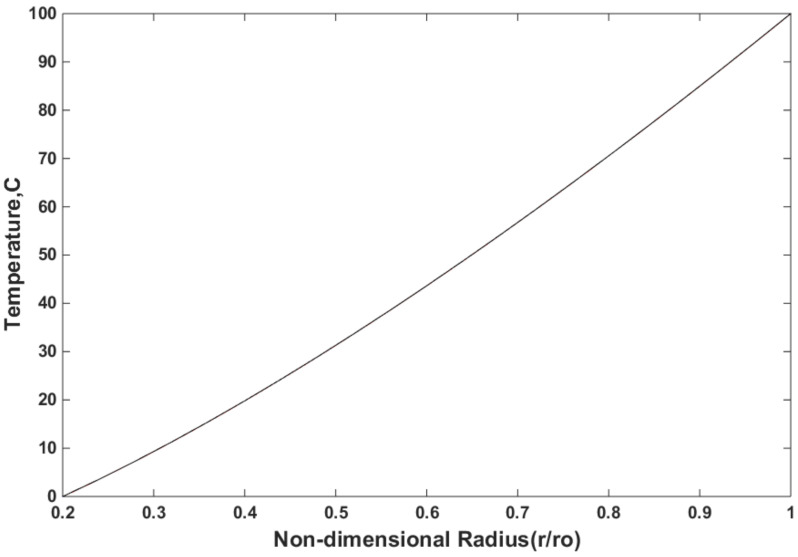
Temperature distribution using three methods for $m_{\mathrm{YM}}\;=\;0.3539$, $m_{\mathrm{TE}}\;=\;-0.23$, and $m_{\mathrm{TC}}\;=\;-1.3575$.

At the inner surface, the temperature was zero (T = 0 °C); at the outer surface, the temperature was 100 °C (T = 100 °C). Figure 5 shows the temperature distribution of the FG disk, which was determined by the three mentioned methods, namely the in-house finite element program, ANSYS, and analytical solution. Figure 5 shows that the values of $m_{\mathrm{YM}}\;=\;0.3539$, $m_{\mathrm{TE}}\;=\;-0.23$, and $m_{\mathrm{TC}}\;=\;-1.3575$ were used. As observed, the results were compared and demonstrate the accuracy of the FEMs used in this study. Figure 5 shows that the results presented by the in-house code compared well with the other two methods, and the accuracy of the in-house code was also tested.

**Figure 6:**
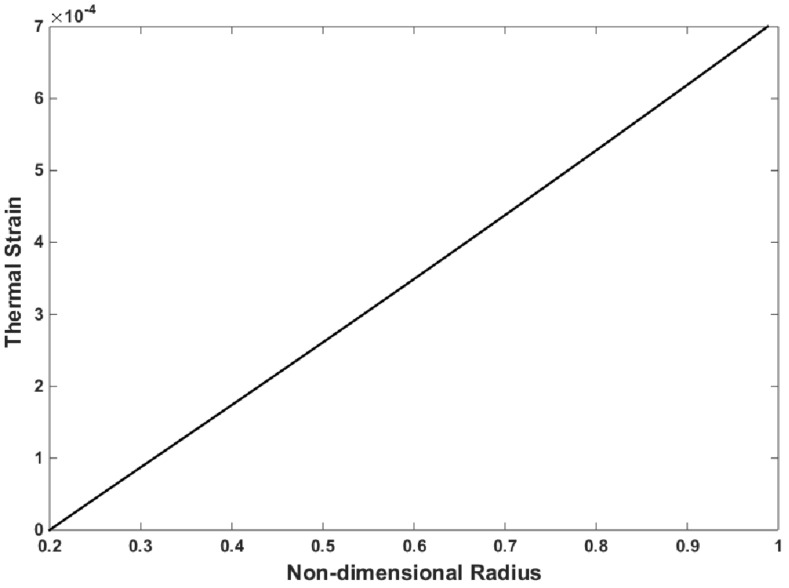
Thermal strain of the FG disk versus the non-dimensional radius for $m_{\mathrm{YM}}\;=\;0.3539$, $m_{\mathrm{TE}}\;=\;-0.23$, and $m_{\mathrm{TC}}\;=\;-1.3575$.

**Figure 7:**
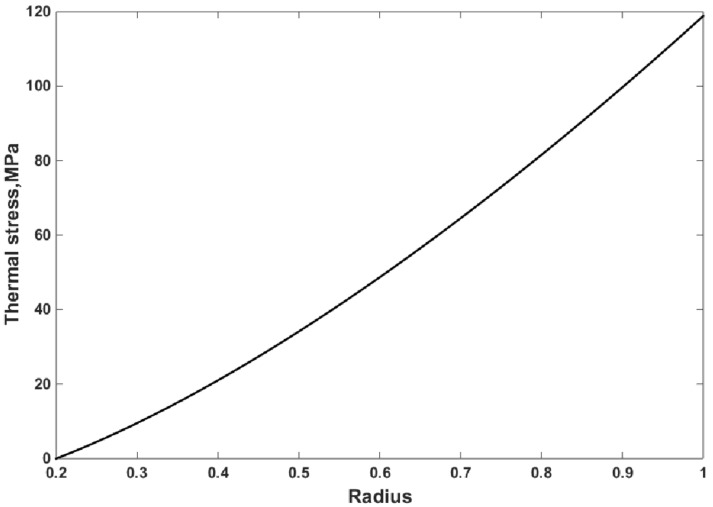
Thermal stress of the FG disk versus the non-dimensional radius for $m_{\mathrm{YM}}\;=\;0.3539$, $m_{\mathrm{TE}}\;=\;-0.23$, and $m_{\mathrm{TC}}\;=\;-1.3575$.

The thermal strain and thermal stress of the FG disk are presented in Figure 6 and Figure 7, respectively. The thermal strain and thermal stress were assessed using the in-house finite element program. The thermal strain and stress have the same trend that exists for temperature along the radius. As expected, and observed in Figure 6 and Figure 7, the maximum values of thermal strain and thermal stress were obtained at the outer surface, where the maximum temperature was applied.

### 5.3. Material Gradation Parametric Study

**Figure 8:**
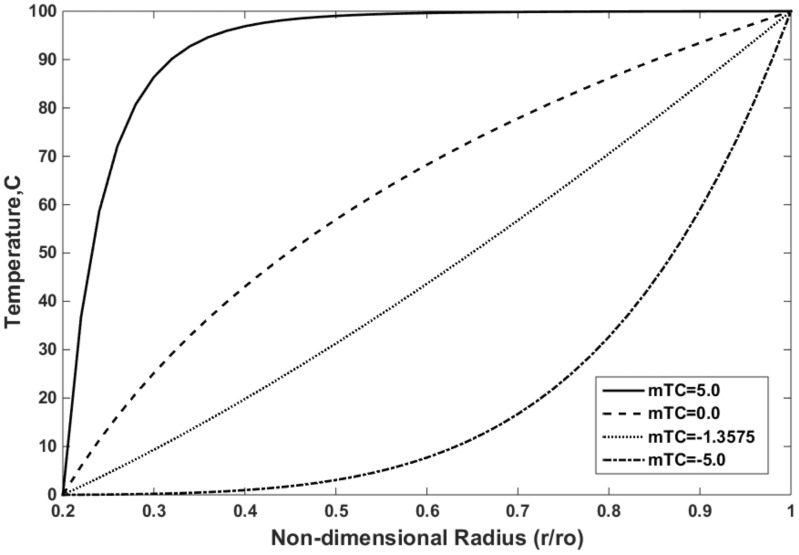
Effect of the $m_{\mathrm{TC}}$ on the temperature distribution in FGMs for $m_{\mathrm{YM}}\;=\;0.3539$ and $m_{\mathrm{TE}}\;=\;-0.23$.

**Figure 9:**
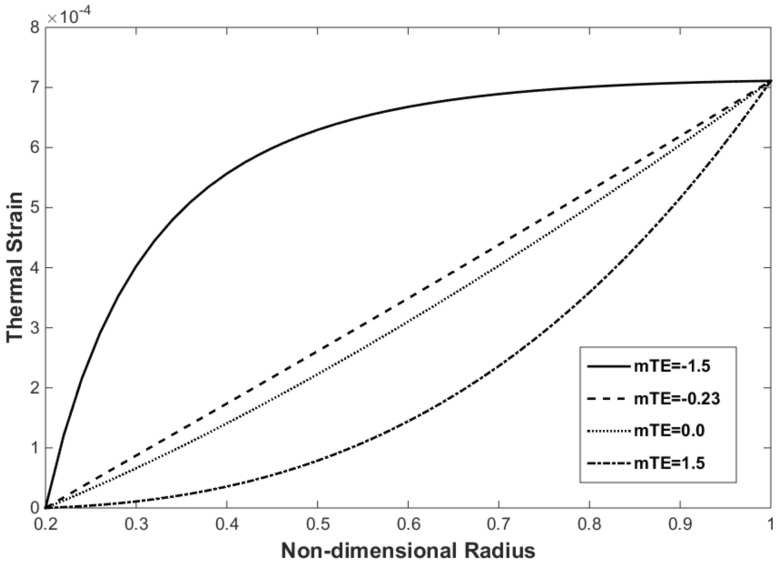
Effect of the $m_{\mathrm{TE}}$ on the thermal strain distribution for $m_{\mathrm{YM}}\;=\;0.3539$ and $m_{\mathrm{TC}}\;=\;-1.3575$.

**Figure 10:**
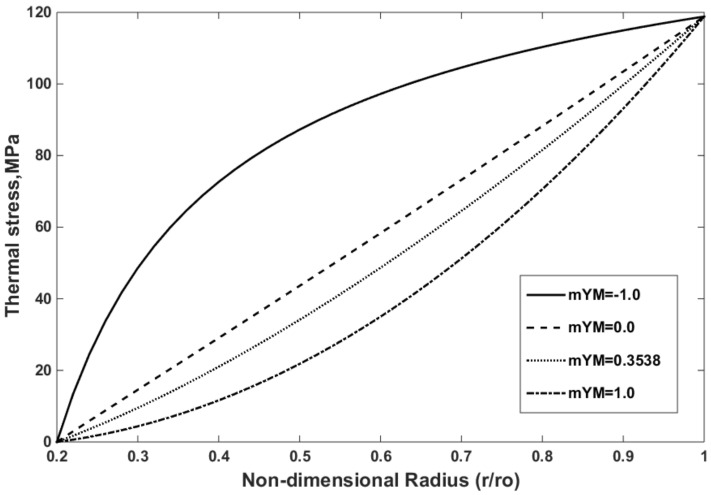
Effect of the $m_{\mathrm{YM}}$ on the thermal stress distribution for $m_{\mathrm{TE}}\;=\;-0.23$ and $m_{\mathrm{TC}}\;=\;-1.3575$.

The effects of $m_{\mathrm{TC}}$, $m_{\mathrm{TE}}$, and $m_{\mathrm{YM}}$ were investigated on the distributions of the temperature, thermal strain, and thermal stress, respectively. The in-house FEM was used, and the results are shown in Figure 8, Figure 9 and Figure 10. The effect of the $m_{\mathrm{TC}}$ on the temperature distribution is shown in Figure 8. The temperature from zero at the inner surface rises to 100 °C at the outer surface of the FGMs. The thermal conductivity coefficient decreases with increasing $m_{\mathrm{TC}}$ (Figure 4a). As observed, the temperature distribution decreases with the decrease in $m_{\mathrm{TC}}$. This trend can be justified following Equation (14), whereby the temperature decreases with the increasing thermal conductivity coefficient. Notably, $m_{\mathrm{TC}}\;=\;0.0$ is attributed to the non-FGM and when the thermal conductivity is distributed uniformly along the radius.

The effect of the $m_{\mathrm{TE}}$ on the thermal strain distribution is shown in Figure 9. The thermal strain decreases with increasing $m_{\mathrm{TE}}$. As observed in Figure 4b, the thermal expansion coefficient decreases with increasing $m_{\mathrm{TE}}$ along the radius in the FG rotating disks. This trend helps decrease the thermal strain with the increase in $m_{\mathrm{TE}}$ following Equation (27). The thermal expansion coefficient is constant along the radius when $m_{\mathrm{TE}}\;=\;0.0,$ but this value does not correspond to the non-FGM because other material properties such as Young’s modulus and coefficient of thermal conductivity vary along the radius.

The effect of the $m_{\mathrm{YM}}$ on the thermal stress distribution is shown in Figure 10. The thermal stress decreases with the increasing $m_{\mathrm{YM}}$. As observed in Figure 4c, the Young’s modulus decreases with the increasing $m_{\mathrm{YM}}$ along the radius in the FG rotating disks. This phenomenon helps decrease the thermal stress with the increase in $m_{\mathrm{YC}}$ following Equation (28). The Young’s modulus is constant along the radius when $m_{\mathrm{YM}}\;=\;0.0,$ but this value does not correspond to the non-FGM because other properties such as the thermal conductivity and thermal expansion coefficients vary along the radius.

The in-house finite element program performs a post-processing analysis in a more efficient and convenient manner than through a simulation in a finite element software program such as ANSYS. For example, an optimization analysis can be carried out to minimize the thermal strain and stress while the stiffness of the plate is maintained. Such an algorithm can be used to calculate the thermal strain and stress, as well as the stiffness, for various ranges of material properties in an FG disk. Appropriate criteria to select the most suitable properties can be applied to present the material properties.

## 6. Conclusions

In this study, the temperature, thermal stress, and strain distributions of an FG disk were determined under an applied thermal condition. The material properties of the FG disk varied along the radial direction following the power law distribution. The inner and outer surfaces of the disk were assumed to be fully metal and fully ceramic, respectively. Three methods were used, namely (i) an in-house finite element program, (ii) the APDL, and (iii) an analytical solution to present the temperature distribution and compare the results for the sake of validation. The thermal conductivity in an FGM varies along an element when using the in-house FE code in contrast with using a commercial FE software when the thermal conductivity is constant within an element. The thermal strain and thermal stress were determined using the in-house finite element program. The temperature distributions obtained using these methods were exactly the same. Thus, in the steady thermal analysis of the FG rotating disk, these three methods are worthwhile because the effect of every material gradation parameter on the results can be investigated when the material properties within an element vary. These findings can lead to improved designs in the future for the fabrication of FGMs subject to thermal loads. The in-house code can be used to perform an optimization analysis to minimize the thermal strain and stress while the stiffness of the plate is maintained. Several numerical examples can be run in a short time to identify the optimized values under various thermal conditions.

## Figures and Tables

**Table 1:** Material properties of the inner and outer surfaces [8].

Material Properties	$E\;\left(GPa\right)$	υ	$\alpha \;\left(\frac{1}{K}\right)$	$K\;\left(\frac{W}{mK}\right)$
Ceramic (outer surface)	117.0	0.3	$7.11\;\times \;10^{-6}$	2.036
Metal (inner surface)	66.2	0.3	$10.3\;\times \;10^{-6}$	18.1

**Table 2:** FG gradation material properties.

$K_{•}$	$m_{TC}$	$\alpha_{•}$	$m_{TE}$	$E_{•}$	$m_{YM}$
2.036	−1.3575	$7.11\;\times \;10^{-6}$	−0.23	117	0.3539

## Data Availability

Not applicable.
